# The Pacific Medical and Surgical Journal

**Published:** 1858-04

**Authors:** 


*The Pacific Medical and Surgical Journal*, Edited and published by John B. Trask, M. D., and David Wooster, M. D.—San Francisco.

This is a new competitor for professional favor. It comes from San Francisco. The January number which is before us, contains several very excellent articles, and two lithographic plates in the best style of the art. The whole getting up of the Journal is good, and we wish it that large measure of success it so richly deserves.

